# Optimal Decision Rules for Biomarker-Based Subgroup Selection for a Targeted Therapy in Oncology

**DOI:** 10.3390/ijms160510354

**Published:** 2015-05-07

**Authors:** Johannes Krisam, Meinhard Kieser

**Affiliations:** Institute of Medical Biometry and Informatics, University of Heidelberg, INF 305, D-69120 Heidelberg, Germany; E-Mail: meinhard.kieser@imbi.uni-heidelberg.de

**Keywords:** adaptive designs, subgroup selection, personalized medicine, targeted therapy, biomarker

## Abstract

Throughout recent years, there has been a rapidly increasing interest regarding the evaluation of so-called targeted therapies. These therapies are assumed to show a greater benefit in a pre-specified subgroup of patients—commonly identified by a predictive biomarker—as compared to the total patient population of interest. This situation has led to the necessity to develop biostatistical methods allowing an efficient evaluation of such treatments. Among others, adaptive enrichment designs have been proposed as a solution. These designs allow the selection of the most promising patient population based on an efficacy analysis at interim and restricting recruitment to these patients afterwards. As has recently been shown, the performance of the applied interim decision rule in such a design plays a crucial role in ensuring a successful trial. In this work, we investigate the situation when the primary outcome of the trial is a binary variable. Optimal decision rules are derived which incorporate the uncertainty about the treatment effects. These optimal decision rules are evaluated with respect to their performance in an adaptive enrichment design in terms of correct selection probability and power, and are compared to proposed *ad hoc* decision rules. Our methods are illustrated by means of a clinical trial example.

## 1. Introduction

Throughout the recent years, triggered by an increasingly more profound understanding of disease mechanisms, clinical researchers have come to the conclusion that the assumption of a homogenous treatment effect throughout the patient population of interest does not always hold true. Instead, it has become known that there are subgroups of patients which may have a larger benefit as compared to the total population. Usually, these subgroups are identified with an (often binary) biomarker, and patients are tested for their biomarker status with a suitable bioassay. We here can distinguish between two types of biomarkers: in case that a biomarker can be used to predict the most likely prognosis of an individual patient, the biomarker is called “prognostic”. If, however, a biomarker is likely to predict the response to a specific therapy, it is called “predictive”, see, e.g., [1]. There are also many cases where a biomarker shows both predictive and prognostic properties.

There exist several examples of therapies which have only been approved for a specific subset of patients identified by a predictive biomarker, such as human epidermal growth factor receptor 2 (HER2) overexpression for the treatment of breast cancer patients, Kirsten rat sarcoma viral oncogene homolog (KRAS) mutations in patients with colorectal cancer, and epidermal growth factor receptor (EGFR) mutations for patients with non-small cell lung cancer [1].

Until recently, the most common approach to evaluate potentially targeted therapies was to identify the most promising subgroup within an exploratory phase II trial and then to investigate this subgroup within a subsequent confirmatory phase III trial. However, this approach has the disadvantage that the data obtained in the exploratory phase II trial cannot be incorporated into the proof of efficacy which can only be claimed based on the data from the confirmatory trial. So-called adaptive enrichment designs, which have recently been proposed as an alternative (see, e.g., [2–13]), do not show this disadvantage. They allow the possibility to select the target population mid-trial at an interim analysis to subsequently investigate the most promising target population, and finally to combine the data from both stages of the trial for the proof of efficacy. There exists a broad range of designs for biomarker-based trials. For an overview we refer to [1]. The investigation of one or more target populations within a single trial yields the problem of multiple testing. In order to control the nominal significance level, adaptive enrichment designs address this issue by incorporating adjustment methods for multiple testing.

Since it crucially influences the properties and outcome of the trial, the role of the applied interim decision rule should not be undervalued. However, there exists only very sparse literature on the impact of the applied interim decision rule on design properties such as power, type I error rate or probability for a correct selection. As has been shown recently for the case of a normally distributed outcome [14,15], the role of the applied decision rule is vital for a clinical trial with subgroup selection. Hence, this article aims to further investigate the role of the applied decision rule in an adaptive enrichment design in case of a binary outcome variable, which has not yet been covered in the literature. Another important issue concerning the determination of a decision rule with desirable properties is the generally common uncertainty about treatment effects. When prior knowledge about treatment effects can be modeled by prior distributions and the sustained loss for a false decision can be quantified by means of a loss function, it is possible to determine optimal decision rules which minimize the expected loss. In this article, we derive such optimal rules, discuss their properties and investigate their performance in an adaptive enrichment trial by means of a simulation study.

The article is structured as follows: Section 2 introduces the test problem and some notation. Section 3 presents the adaptive enrichment design and the related testing procedure. Optimal decision rules are derived in Section 4 for a wide range of scenarios. Section 5 investigates the impact of these rules in terms of power and probability for a correct selection within the proposed trial design by means of a simulation study. The practical utility of our proposed rules is illustrated in Section 6 by a clinical trial example. Finally, we conclude with a discussion in Section 7.

## 2. Notation and General Considerations

Throughout this article, a parallel group randomized controlled trial is considered, where an experimental treatment *T* is compared to a control treatment *C*. The participants of the trial stem from a total patient population *G*_0_ which contains a subset *G*_1_ and a complementary subset *G*_2_ := *G*_0_ *\ G*_1_. Patients from *G*_1_ are expected to have a greater benefit from the investigated therapy and are identified by a biomarker. Biomarker status is assessed by a bioassay and patients with a positive bioassay outcome are identified as biomarker-positive. Let us assume that that treatment allocation is balanced and both treatment groups are assumed to contain an identical proportion of biomarker-postive patients π. The primary outcome is a binary variable taking values 0 and 1, where 1 stands for a favorable und 0 for an unfavorable event. Let *p_T_*_1_ and *p_C_*_1_ be the rates of success for subgroup *G*_1_ in the treatment and control group, respectively, and *p_T_*_2_ and *p_C_*_2_ be the respective treatment success rates in the complementary subset *G*_2_. The efficacy measures of our trial are the differences in event rates ∆_1_ := *p_T_*_1_ − *p_C_*_1_, ∆_2_ := *p_T_*_2_ − *p_C_*_2_ and the difference in event rates in the total population ∆_0_ = π∆_1_ + (1 − π)∆_2_. The one-sided global null and alternative hypothesis for our trial are then:

$$H_{0}:\Delta_{0}\leq 0\cap \Delta_{1}\leq 0\;vs.\;H_{1}:\Delta_{0}>0\cup \Delta_{1}>0$$

This global test problem consists of two local test problems for the respective populations of interest:

$$\begin{array}{l} H_{0}^{\left(0\right)}:\Delta_{0}\leq 0\;vs.\;H_{1}^{\left(0\right)}:\Delta_{0}>0\;\text{and}\\ H_{0}^{\left(1\right)}:\Delta_{1}\leq 0\;vs.\;H_{1}^{\left(1\right)}:\Delta_{1}>0 \end{array}$$

In order to properly control the family wise error rate in this situation of multiple testing, a closed testing procedure will be applied. This means that a local null hypothesis, e.g., $H_{0}^{\left(0\right)}$, can only be rejected at level *a*, if the global hypothesis *H*_0_ which implies $H_{0}^{\left(0\right)}$ is also rejected at level *a*. Further details on the testing procedure will be described in the following section.

The outcome variables are assumed to be independent with:

$$\begin{array}{c} {\left(X_{i}^{T1}\right)}_{i}{∼}_{\mathrm{iid}}Ber\left(p_{T1}\right),\;{\left(X_{j}^{C1}\right)}_{j}{∼}_{\mathrm{iid}}Ber\left(p_{C1}\right)\\ {\left(X_{k}^{T2}\right)}_{k}{∼}_{\mathrm{iid}}Ber\left(p_{T2}\right),\;{\left(X_{l}^{C2}\right)}_{l}{∼}_{\mathrm{iid}}Ber\left(p_{C2}\right) \end{array}$$

where *Ber*(*p*) denotes the Bernoulli distribution which is the probability distribution of a random variable taking the value 1 with a success probability of *p* and value 0 with a failure probability of *q* = 1−*p*.

## 3. Trial Design and Adaptive Testing Procedure

In this article, we consider a two-stage adaptive enrichment trial which was proposed for a time-to-event outcome by Jenkins *et al*. [7] which can be transferred to the setting of a binary outcome variable by appropriate modifications. The proposed design consists of two trial stages *I* and *II* with an *a* priori fixed sample size for both stages of *n* patients per treatment group, which may, however, change after an interim analysis after stage *I*. At this point, an interim decision concerning the target population for the subsequent stage of the trial has to be made. The decision is based on the observed event rates in the first stage of the trial and incorporates four possible options for the second stage and, accordingly, the final analysis:

(1) *G*_0_ and *G*_1_ are selected as co-primary target populations
(2) *G*_0_ is selected as the only target population
(3) *G*_1_ is selected as the only target population
(4) The trial is stopped for futility

**Figure 1:**
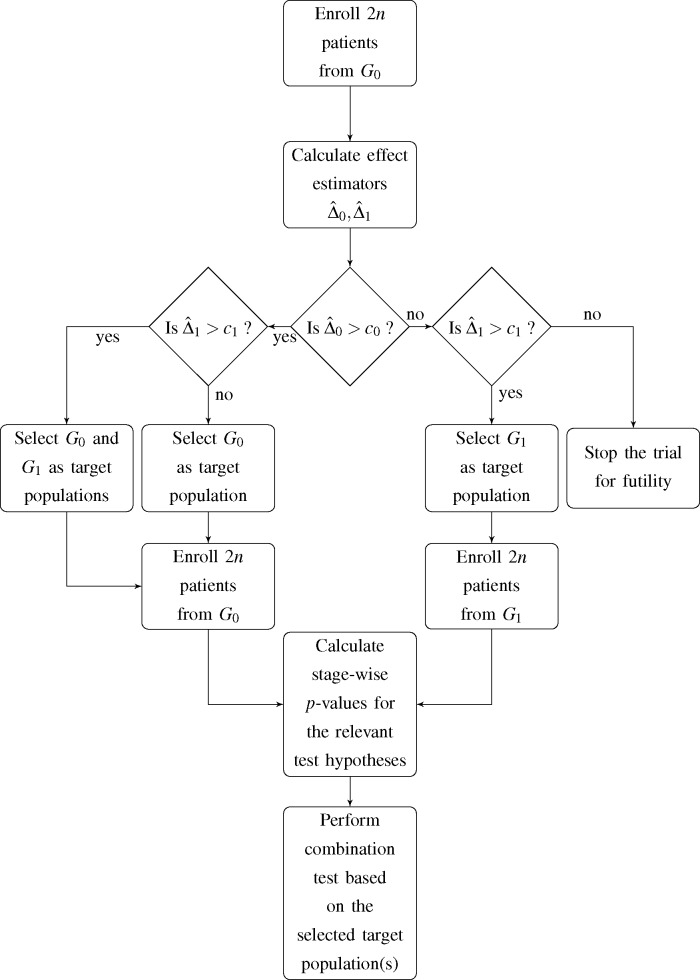
Flowchart of the considered adaptive two-stage design.

Jenkins *et al*. [7] expressed the decision rule in terms of the estimated hazard ratio in the total population and subgroup. Since we deal with a binary outcome variable, we choose the observed difference in event rates in both *G*_0_ and *G*_1_ as decision criterion. Hence, if the observed difference in event rates within population *G_i_*, ${\hat{\Delta }}_{i}$, does not exceed the pre-specified decision threshold *c_i_*, *i* = 0,1, the respective population is dropped as a target population for the second stage of the trial. A flowchart of the proposed trial design is shown in Figure 1.

### 3.1. First Trial Stage

Let

$$\begin{array}{l} {\hat{p}}_{T0}^{I}:=\frac{1}{n}\left(\sum_{i=1}^{\pi n}X_{i}^{T1}+\sum_{k=1}^{(1-\pi )n}X_{k}^{T2}\right),{\hat{p}}_{C0}^{I}:=\frac{1}{n}\left(\sum_{j=1}^{\pi n}X_{j}^{C1}+\sum_{l=1}^{(1-\pi )n}X_{l}^{T2}\right),\\ \;{\hat{p}}_{0}^{I}:=\frac{1}{2n}\left(\sum_{i=1}^{\pi n}X_{i}^{T1}+\sum_{k=1}^{(1-\pi )n}X_{k}^{T2}+\sum_{j=1}^{\pi n}X_{j}^{C1}+\sum_{l=1}^{(1-\pi )n}X_{l}^{T2}\right)\\ {\hat{p}}_{T1}^{I}:=\frac{1}{\pi n}\sum_{i=1}^{\pi n}X_{i}^{T1},\;{\hat{p}}_{C1}^{I}:=\frac{1}{\pi n}\sum_{j=1}^{\pi n}X_{j}^{T1},\;{\hat{p}}_{1}^{I}:=\frac{1}{2\pi n}\left(\sum_{i=1}^{\pi n}X_{i}^{T1}+\sum_{j=1}^{\pi n}X_{j}^{T1}\right) \end{array}$$

be the event rate estimators of the first stage of the trial. The treatment effect estimators are then:

$${\hat{\Delta }}_{0}:={\hat{p}}_{T0}^{I}-{\hat{p}}_{C0}^{I},\;{\hat{\Delta }}_{1}:={\hat{p}}_{T1}^{I}-{\hat{p}}_{C1}^{I}$$

Test statistics $\left(Z_{0}^{I},Z_{1}^{I}\right)$ and stage-wise *p*-values for the first stage of the trial, $p_{0}^{I}$, $p_{1}^{I}$ $p_{01}^{I}$ are defined as:

$$\begin{array}{l} Z_{0}^{I}:={\hat{\Delta }}_{0}/\sqrt{{\hat{p}}_{0}^{I}\left(1-{\hat{p}}_{0}^{I}\right)\left(2/n\right)},\;Z_{1}^{I}:={\hat{\Delta }}_{1}/\sqrt{{\hat{p}}_{1}^{I}\left(1-{\hat{p}}_{1}^{I}\right)\left(2/\left(\pi n\right)\right)}\\ p_{0}^{I}:=1-\Phi \left(Z_{0}^{I}\right),\;p_{1}^{I}:=1-\Phi \left(Z_{1}^{I}\right),\;p_{01}^{I}:=\min\left[2\min\left\{p_{0}^{I},p_{1}^{I}\right\},\max\left\{p_{0}^{I},p_{1}^{I}\right\}\right] \end{array}$$

where Φ denotes the distribution function of the standard normal distribution. The *p*-value $p_{01}^{I}$ corresponds to the global null hypothesis *H*_0_ and is a multiplicity-corrected *p*-value according to the method of Hochberg [16]. We chose the *z*-test for difference in proportions to test the trial hypotheses due to their one-sided nature. It should be noted that the same method is also used in one of the most frequently used software for planning and analysis of adaptive enrichment designs, ADDPLAN™ [17].

### 3.2. Second Trial Stage

In case both total population and subgroup are selected as co-primary target population or only the total population is chosen for the second stage of the trial, this will yield event rate estimators:

$$\begin{array}{l} {\hat{p}}_{T0}^{II}:=\frac{1}{n}\left(\sum_{i=\pi n+1}^{2\pi n}X_{i}^{T1}+\sum_{k=(1-\pi )n+1}^{2(1-\pi )n}X_{k}^{T2}\right),\;{\hat{p}}_{C0}^{II}:=\frac{1}{n}\left(\sum_{i=\pi n+1}^{2\pi n}X_{j}^{C1}+\sum_{l=(1-\pi )n+1}^{2(1-\pi )n}X_{l}^{T2}\right),\\ \;{\hat{p}}_{0}^{II}:=\frac{1}{2n}\left(\sum_{i=\pi n+1}^{2\pi n}X_{i}^{T1}+\sum_{k=(1-\pi )n+1}^{(1-\pi )n}X_{k}^{T2}+\sum_{i=\pi n+1}^{2\pi n}X_{j}^{C1}+\sum_{l=(1-\pi )n+1}^{2(1-\pi )n}X_{l}^{T2}\right),\;\\ {\hat{p}}_{T1}^{II}:=\frac{1}{\pi n}\sum_{i=\pi n+1}^{2\pi n}X_{i}^{T1}\;{\hat{p}}_{C1}^{II}:=\frac{1}{\pi n}\sum_{j=\pi n+1}^{2\pi n}X_{j}^{T1},\;{\hat{p}}_{1}^{II}:=\frac{1}{2\pi n}\left(\sum_{i=\pi n+1}^{2\pi n}X_{i}^{T1}+\sum_{j=\pi n+1}^{2\pi n}X_{j}^{T1}\right) \end{array}$$

and test statistics $\left(Z_{0}^{II},Z_{1}^{II}\right)$ and *p*-values $p_{0}^{II},p_{1}^{II},p_{01}^{II}$ will be calculated analogously as for the first trial stage.

In case that the subgroup is chosen as target population at interim, 2*n* bioassay-positive patients will be enrolled for the second stage of the trial. Hence, the event rate estimators now are:

$$\begin{array}{l} {\hat{p}}_{T1}^{II}:=\frac{1}{n}\sum_{i=\pi n+1}^{\pi n+n}X_{i}^{T1},\;{\hat{p}}_{C1}^{II}:=\frac{1}{n}\sum_{j=\pi n+1}^{\pi n+n}X_{j}^{T1}\\ \;{\hat{p}}_{1}^{II}:=\frac{1}{2n}\left(\sum_{i=\pi n+1}^{\pi n+n}X_{i}^{T1}+\sum_{j=\pi n+1}^{\pi n+n}X_{j}^{T1}\right) \end{array}$$

This yields the following test statistics and *p*-value:

$$Z_{1}^{II}:=\frac{{\hat{p}}_{T1}^{II}-{\hat{p}}_{C1}^{II}}{\sqrt{{\hat{p}}_{1}^{II}\left(1-{\hat{p}}_{1}^{II}\right)\left(2/n\right)}},\;p_{1}^{II}:=1-\Phi \left(Z_{1}^{II}\right)$$

In analogy to [7], for the co-primary case, *i.e.*, when both populations are considered as target populations, the hypotheses *H*_0_ $H_{0}^{\left(0\right)}$, $H_{0}^{\left(1\right)}$ will be assessed via the following combination test statistics:

$$\begin{array}{l} Z_{01}:=\frac{1}{\sqrt{2}}\Phi^{-1}\left(1-p_{01}^{I}\right)+\frac{1}{\sqrt{2}}\Phi^{-1}\left(1-p_{01}^{II}\right)\\ Z_{0}:=\frac{1}{\sqrt{2}}\Phi^{-1}\left(1-p_{0}^{I}\right)+\frac{1}{\sqrt{2}}\Phi^{-1}\left(1-p_{0}^{II}\right)\\ Z_{1}:=\frac{1}{\sqrt{2}}\Phi^{-1}\left(1-p_{1}^{I}\right)+\frac{1}{\sqrt{2}}\Phi^{-1}\left(1-p_{1}^{II}\right) \end{array}$$

Here, we chose the reasonable approach to weigh the stage-wise *p*-values equally due to the identical sample sizes across stages. The combined test statistics in case of selecting the total population as the only target population are only slightly different:

$$\begin{array}{l} Z_{01}:=\frac{1}{\sqrt{2}}\Phi^{-1}\left(1-p_{01}^{I}\right)+\frac{1}{\sqrt{2}}\Phi^{-1}(1-p_{0}^{II})\\ Z_{0}:=\frac{1}{\sqrt{2}}\Phi^{-1}\left(1-p_{0}^{I}\right)+\frac{1}{\sqrt{2}}\Phi^{-1}(1-p_{0}^{II}) \end{array}$$

Since the hypothesis $H_{0}^{\left(1\right)}$ was dropped at interim, $p_{0}^{II}$ is chosen as the *p*-value for the global hypothesis *H*_0_ at the second trial stage, see [7].

If enrollment is restricted to the subgroup only after the interim analysis, we have:

$$\begin{array}{l} Z_{01}:=\frac{1}{\sqrt{2}}\Phi^{-1}\left(1-p_{01}^{I}\right)+\frac{1}{\sqrt{2}}\Phi^{-1}\left(1-p_{1}^{II}\right)\\ Z_{1}:=\sqrt{\frac{1}{1+\pi }}\Phi^{-1}\left(1-p_{1}^{I}\right)+\sqrt{\frac{1}{1+\pi }}\Phi^{-1}\left(1-p_{1}^{II}\right) \end{array}$$

Note that $p_{1}^{II}$ is chosen as the *p*-value for the global null hypothesis at the second stage and that the weights for $H_{0}^{\left(II\right)}$ are adjusted in order to properly reflect the different sample sizes of patients from *G*_1_ enrolled in stages *I* and *II*.

Finally, efficacy of the treatment will be analyzed by first testing *H*_0_. If the null hypothesis can be rejected at one-sided significance level *α*, $H_{0}^{\left(0\right)}$ and $H_{0}^{\left(1\right)}$ will be tested at the same *a*-level if the respective hypothesis was not dropped at interim. Note that a claim of efficacy in either of the target populations is only valid if one of the local null hypotheses can be rejected but cannot be justified when only *H*_0_ can be rejected.

## 4. Optimization of Decision Rules

When planning a clinical trial with the design described above, the decision thresholds (*c*_0_, *c*_1_) have to be chosen carefully. Usually, they should reflect considerations in which situation a treatment effect is meaningful enough to pursue investigation of the therapy in the respective study population. The determination of decision boundaries, however, may be difficult when the magnitude of the treatment effects is uncertain, which is usually the case. If the uncertainty about treatment effects can be modeled in terms of prior distributions, it is possible to determine an optimal decision rule. The optimality criterion comes from a so-called loss function, which has to be pre-specified and yields a penalization in case of a false decision. Then, the optimal decision rule is the one that minimizes the expected loss which sometimes is also called Bayes risk (see, e.g., [18]). In order to determine whether a decision is correct or not, it is necessary to specify two relevance thresholds τ_0_ and τ_1_. If the actual treatment effect ∆*_i_* exceeds *t_i_*, it would be correct to further investigate population *G_i_* and, vice versa, if ∆*_i_* ≤ τ*_i_*, it would be correct to stop enrolling patients from *G_i_* for the second stage of the trial, *i* = 0,1.

### 4.1. Derivation of Optimal Decision Thresholds

In order to properly model the Bayes risk, we first need to derive some distributional properties of our effect measures, the rate difference estimators from the first stage of the trial:

$${\hat{\Delta }}_{0}={\hat{p}}_{T0}^{I}-{\hat{p}}_{C0}^{I},\;{\hat{\Delta }}_{1}={\hat{p}}_{T1}^{I}-{\hat{p}}_{C1}^{I}$$

with expectations, variances and covariance (for derivation, see Appendix A.1):

$$\begin{array}{l} E\left[{\hat{\Delta }}_{0}\right]=\pi (p_{T1}-p_{C1})+(1-\pi )(p_{T2}-p_{C2}),\;E\left[{\hat{\Delta }}_{1}\right]=p_{T1}-p_{C1},\\ \mathrm{Var}\left[{\hat{\Delta }}_{0}\right]=\frac{\pi p_{T1}(1-p_{T1})+p_{C1}(1-p_{C1}))+(1-\pi )(p_{T2}(1-p_{T2})+p_{C2}(1-p_{C2}))}{n},\\ \mathrm{Var}\left[{\hat{\Delta }}_{0}\right]=\frac{p_{T1}(1-p_{T1})+p_{C1}(1-p_{C1})}{\pi n},\;\mathrm{Cov}\left[{\hat{\Delta }}_{0},{\hat{\Delta }}_{1}\right]=\frac{p_{T1}(1-p_{T1})+p_{C1}(1-p_{C1})}{n} \end{array}$$

Now let Y be the two-dimensional space of realizations of the bivariate estimator $\left({\hat{\Delta }}_{0},{\hat{\Delta }}_{1}\right)$. Let $D:=\left\{d_{\left(c_{0},c_{1}\right)},\;\left(c_{0},c_{1}\right)\in {\left[-1,1\right]}^{2}\right\}$ denote a set of decision rules with $d_{\left(c_{0},c_{1}\right)}:Y\to A$, such that:

$$d_{\left(c_{0},c_{1}\right)}\left({\tilde{y}}_{0},{\tilde{y}}_{1}\right)=\left(1_{\left\{{\tilde{y}}_{0}>c_{0}\right\}}\left({\tilde{y}}_{0}\right),1_{\left\{{\tilde{y}}_{1}>c_{1}\right\}}\left({\tilde{y}}_{1}\right)\right),\;1_{\left\{y>b\right\}}\left(y\right)=\{\begin{array}{ll} 1 & \mathrm{if}\;y>b,\\ 0 & \text{else}. \end{array}$$

In our case, the space of possible actions $A={\left\{0,1\right\}}^{2}$ contains the following four elements: (1, 1) denotes the co-primary case, (1, 0) leads us to drop the subgroup as a target population, (0, 1) means that solely patients from the subgroup are enrolled for the second stage of the trial and (0, 0) stands for stopping the trial for futility. We assume that continuation of the total population as a target population is desirable if the actual treatment effect exceeds the pre-specified relevance threshold *τ*_0_ and, accordingly, selection of the subgroup as target population is desired if the true effect in the subgroup exceeds *τ*_1_. Otherwise, discontinuation of enrollment from the respective population is desired.

We now have to quantify the sustained loss given a false decision has occurred in order to find a decision rule minimizing the expected loss. Hence, we have to define both a loss function and to model our knowledge about event rates in the respective sub- and treatment groups by prior distributions. A frequently employed loss function is the so-called quadratic loss function, which is, mainly due to its simplicity, the most popular loss function for decision theoretic approaches and which is commonly used for sequential trial designs (see, e.g., [19]). In our case, it yields a loss which is the squared difference between the treatment effects ∆*_i_* and the relevance thresholds *τ_i_*, *i* = 0,1, in case of a false decision (for the mathematical definition of the loss function, see Appendix A.2).

Throughout this article, π*_T_*_1_, π*_C_*_1_, π*_T_*_2_, π*_C_*_2_ are defined as the prior random variables for the respective event rates *p_T_*_1_, *p_C_*_1_, *p_T_*_2_, *p_C_*_2_ in the treatment groups *T*, *C* and subgroups *G*_1_, *G*_2_, which are not to be mixed up with the subgroup prevalence π. We assume that these priors follow independently distributed continuous uniform distributions with:

$$\pi_{ij}∼U_{\left[a_{ij},b_{ij}\right]},\;\text{with}\;0\leq a_{ij}<b_{ij}\leq 1,\;i=T,C,j=1,2$$

However, it is also possible to apply other meaningful prior distributions, such as a triangular or a (truncated) normal distribution.

The Bayes risk *r* can now be modeled by incorporating the distributional properties of the employed priors and effect estimators $\left({\hat{\Delta }}_{0},{\hat{\Delta }}_{1}\right)$ conditional on π*_ij_*, *i* = *T*, *C*, *j* = 1, 2. The optimal decision rule $d_{\left(c_{0}^{*},c_{1}^{*}\right)}$ is the one which yields the minimal Bayes risk, *i.e.*,

$$r\left(d_{\left(c_{0}^{*},c_{1}^{*}\right)}\right)=\underset{d\in D}{\min}r\left(d\right)$$

After calculating the derivative of the Bayes risk (for details, see Appendix A.2), we used *Mathematica* 9.0 [20] for numerical integration and root solving to determine the optimal decision thresholds (program code is provided as Supplementary Information). In order to obtain the results presented in this manuscript, the precision for numerical integration by local adaptive method was set to 8 digits, and 3 digits were chosen for the precision for the numerical root solving procedure. In case the root was found to be out of the bounds of reasonable decision thresholds [−1, 1], the sign of the root, *i.e.*, either −1 or 1, was chosen as optimal decision threshold.

### 4.2. Examples for Optimal Decision Thresholds

In this subsection we provide some optimal decision rules which were derived for some specific parameter situations. In the following, we consider three different prior situations:

(1) The biomarker is assumed to be predictive for treatment effect with prior knowledge about treatment effect modeled as:
  $$\pi_{T1}∼U_{[0.3,0.6],}\;\pi_{T2},\pi_{C1},\pi_{C2}{∼}_{\mathrm{iid}}U_{\left[0.1,0.4\right]}$$
(2) The biomarker is assumed to be predictive and prognostic with prior knowledge about treatment effect modeled as:
  $$\pi_{T1}∼U_{[0.3,0.6],}\;\pi_{C1}∼U_{\left[0.05,0.35\right]},\;\pi_{T2}∼U_{\left[0.2,0.5\right]},\;\pi_{C2}∼U_{\left[0.2,0.5\right]}$$
(3) There is no prior knowledge about the event rates at all, *i.e.*, the prior is non-informative:
  $$\pi_{ij}{∼}_{\mathrm{iid}}U_{\left[0.1\right]},\;i=T,C,j=0,1$$

Optimal decision thresholds were determined for relevance thresholds τ_0_ = 0*:*05 and τ_1_ = 0*:*1. We investigated sample sizes per group and stage starting from *n* = 20 and increasing up to *n* = 200 in steps of 20, for *n* = 300 and *n* = 400. The results are shown in Tables 1–3.

In Tables 1 and 2, it can be observed that given subgroup prevalence π or sample size *n* is small, the optimal decision threshold $c_{1}^{*}$ tends to be relatively low thus favoring a selection of the subgroup at interim. $c_{1}^{*}$ then gradually increases with increasing subgroup prevalence and sample size, and it approaches the relevance threshold τ_1_ = 0.1. In case of a predictive prior, the optimal decision threshold $c_{1}^{*}$ for the total population also approaches the respective relevance threshold *τ*_0_. However, it can be observed in Table 1 that it approaches this value from above in case of π = 0*:*1 and π = 0*:*25 and, in contrast, from below in case of π = 0*:*5. This holds also true for the case of a predictive and prognostic prior, with the exception of the prevalence situation π = 0*:*25, where the optimal decision threshold now approaches *t*_0_ from below. This may be explained by the fact that in case of an increasing prevalence of the subgroup, it may be more desirable to select the total population, since with an increasing subgroup prevalence, the treatment effect ∆_0_ gradually increases too.

Table 3 displays optimal decision thresholds in case of a non-informative prior. One can observe that as compared to the previously discussed cases of prior knowledge, the optimal decision thresholds stick relatively close to the relevance thresholds. All of these thresholds exceed the respective relevance thresholds, but not to a great extent. It can also be observed that the optimal thresholds approach the relevance thresholds with increasing sample size. Interestingly, for an increasing subgroup prevalence, $c_{0}^{*}$ however will depart from *τ*_0_, while on the other hand $c_{1}^{*}$ will approach *τ*_1_.

## 5. Simulation Study

In this section, we compare the performance of the previously derived optimal decision rules to the performance of an *ad hoc* decision rule in terms of statistical power within a simulation study. The decision thresholds are employed in the adaptive enrichment design presented in Section 3. A sample size per group and stage of *n* = 200 was chosen, and the one-sided significance level was set to *a* = 0*:*025. It is assumed that it is desirable to continue with the total population if the actual treatment effect ∆_0_ exceeds *τ*_0_ = 0*:*05, and selection of the subgroup is desired if ∆_1_ > *τ*_1_ = 0*:*1. We chose the following situation for the respective event rates: *p_T_*_1_ = 0*:*45*; p_C_*_1_ = 0.3, *p_T_*_2_ = 0.43, and *p_C_*_2_ = 0.4. Three situations were considered for the subgroup prevalence, namely π = 0.1, 0.25, 0.5. The treatment effect in the subgroup is ∆_1_ = 0.45 − 0.3 = 0.15, and thus, selection of the subgroup is desirable in all considered situations. If π = 0.1, the treatment effect in the total population is ∆_0_ = 0.1 (0.45 – 0.3) + 0.9 (0.43 − 0.4) = 0.042 < *τ*_0_ and, hence, it would be desirable to continue only with the subgroup here. For the situations π = 0.25 and π = 0.5, we have ∆_0_ = 0.06 and ∆_0_ = 0.09, respectively. In these latter two situations, the treatment effect in the total population exceeds the relevance threshold *τ*_0_ = 0.05. Hence, it would be desirable to investigate both target populations during the second stage of the trial.

In the following, we will consider four distinct decision rules:

(a) an *ad hoc* rule based on the relevance thresholds, *i.e.*, (*c*_0_, *c*_1_) = (*τ*_0_, *τ*_1_) = (0.05, 0.1),
(b) an optimal decision rule based on a prior which assumes that the biomarker is predictive, *i.e.*,
  $$\pi_{T1}∼U_{\left[0.3,0.6\right]},\;\pi_{T2},\pi_{C2},\pi_{C2}{∼}_{\mathrm{iid}}U_{\left[0.1,0.4\right]}$$
  which yields the decision thresholds
  - $\left(c_{0}^{*},c_{1}^{*}\right)=\left(0.0543,-0.0369\right)$ for π = 0.1,
  - $\left(c_{0}^{*},c_{1}^{*}\right)=\left(0.0501,0.0525\right)$ for π = 0.25,
  - $\left(c_{0}^{*},c_{1}^{*}\right)=\left(0.0387,0.0785\right)$ for π = 0.5,
(c) an optimal decision rule based on a prior which assumes that the biomarker is predictive and prognostic, *i.e.*,
  $$\pi_{T1}∼U_{\left[0.3,0.6\right]},\;\pi_{C1}∼U_{\left[0.05,0.35\right]},\;\pi_{T2}∼U_{\left[0.2,0.5\right]},\;\pi_{C2}∼U_{\left[0.2,0.5\right]}$$
  which yields the decision thresholds
  - $\left(c_{0}^{*},c_{1}^{*}\right)=\left(0.0541,-0.1333\right)$ for π = 0.1,
  - $\left(c_{0}^{*},c_{1}^{*}\right)=\left(0.0477,0.0176\right)$ for π = 0.25,
  - $\left(c_{0}^{*},c_{1}^{*}\right)=\left(0.0310,0.0645\right)$ for π = 0.5,
(d) an optimal decision rule based on a noninformative prior, *i.e.*,
  $$\pi_{ij}{∼}_{\mathrm{iid}}U_{\left[0,1\right]},\;i=T,C,j=0,1$$
  which yields the decision thresholds
  - $\left(c_{0}^{*},c_{1}^{*}\right)=\left(0.0507,0.1118\right)$ for π = 0.1,
  - $\left(c_{0}^{*},c_{1}^{*}\right)=\left(0.0509,0.1046\right)$ for π = 0.25,
  - $\left(c_{0}^{*},c_{1}^{*}\right)=\left(0.0511,0.1023\right)$ for π = 0.5.

**Figure 2:**
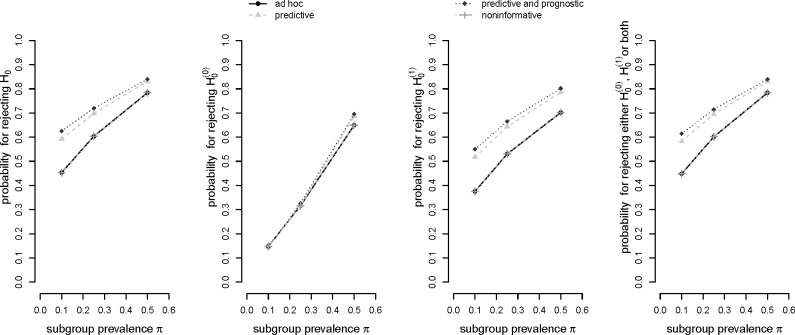
Power in dependence of subgroup prevalence π for *p_T_*_1_ = 0.45*; p_C_*_1_ = 0.3, *p_T_*_2_ = 0.43*; p_C_*_2_ = 0.4, and *n* = 200.

We used Monte Carlo simulations to obtain our results (program code is provided as Supplementary Information) and simulated 1,000,000 data sets per scenario (standard error equals 5·10^−4^ for a rate of 0.5). Figure 2 shows the respective probabilities to

- reject the global null hypothesis *H*_0_,
- reject the global null hypothesis $H_{0}^{\left(0\right)}$,
- reject the global null hypothesis $H_{0}^{\left(1\right)}$,
- reject either $H_{0}^{\left(0\right)}$, $H_{0}^{\left(1\right)}$, or both.

It can be observed in Figure 2 that the *ad hoc* decision rule (a) and the “noninformative” optimal rule (d) yield the same results. Obviously, the decision thresholds in (d) exceed the respective relevance thresholds only by a slight margin which apparently did not yield to a difference in statistical power. In general, decision rule (c) based on a prognostic and predictive prior assumption shows the best performance in terms of probability for a rejection of the global null hypothesis *H*_0_. Decision rule (b) based on a predictive prior assumption only performs slightly worse. This pattern prevails for the probability of rejecting the local null hypothesis $H_{0}^{\left(1\right)}$ as well as the rejection of either one or both of the two local null hypotheses. All four decision rules show a comparable performance concerning the rejection of the local null hypothesis for the total population, $H_{0}^{\left(0\right)}$. For the situation π = 0.5, however, it can be observed that, again, decision rules (b) and (c) slightly outperform the other rules. In all other cases the figure shows that the difference in power between the four rules slightly decreases with increasing subgroup prevalence.

The advantage of decision rules (b) and (c) over rules (a) and (d) may be explained by the fact that they favor the selection of the subgroup for all three prevalence scenarios due to their rather generous decision threshold *c*_1_. Furthermore, the decision thresholds for the total population *c*_0_ are also slightly more generous as compared to rules (a) and (d) for an increasing subgroup prevalence which, accordingly, yields to an advantage in power.

## 6. Application to a Clinical Trial Example

One of the most prominent examples of a targeted therapy is the monoclonal antibody trastuzumab. Treatment with trastuzumab as an add-on combined with chemotherapy has shown to be effective as a breast cancer treatment in patients which overexpress HER2, occurring in about 20% to 30% of all invasive breast cancer carcinoma (see, e.g., [21]). Up to today, trastuzumab has only been approved as a treatment for HER2-positive patients. However, recent evidence has led medical researchers to believe that trastuzumab might also be effective for HER2-negative patients. Currently, there is an ongoing large randomized controlled trial, which investigates the efficacy of trastuzumab in HER2-negative patients (ClinicalTrials.gov Identifier. NCT01548677).

For illustrative purposes, let us assume that trastuzumab has not yet been demonstrated to be effective in neither the total population of breast cancer patients nor in the subgroup, and that a research team plans to investigate its efficacy in terms of 5 year event-free survival. An event is defined as disease recurrence, progression or death from any cause and an adaptive enrichment design is chosen. When planning the trial, the researchers used the results from the NOAH trial, a randomized controlled trial investigating the efficacy of trastuzumab combined with chemotherapy (*n* = 117) versus chemotherapy alone (*n* = 118) within HER2-positive patients [22]. This trial, however, additionally featured a parallel HER2-negative cohort which received neoadjuvant chemotherapy alone (*n* = 99). The 5-year event-free survival rates in this trial were 0.58 (95%-CI = [0.48–0.66]) in the experimental group and 0.43 (95%-CI = [0.43–0.52]) in the control group of HER2-positive patients. The survival rates within the parallel HER2-negative patients receiving chemotherapy only amounted to 0.61 (95%-CI = [0.50–0.70]).

Let us further assume that the researchers select the design proposed in this article with a sample size per group and stage of *n* = 400 and a one-sided significance level of *α* = 0.025 and that the prevalence of HER2-positive patients is π = 0.2. It is furthermore assumed that a selection of the total population would be desired if the treatment effect ∆_0_ exceeds *τ*_0_ = 0.08 and, respectively, it is desirable to select the subgroup as a target population if ∆_1_ > *τ*_1_ = 0.1.

With regard to the interim decision rule, let us consider the following four plausible scenarios.

(a) The trial team decides not to incorporate the knowledge from the NOAH trial but to choose the relevance thresholds *τ*_0_, *τ*_1_ as *ad hoc* decision thresholds, *i.e.*, *c*_0_ = 0.08*; c*_1_ = 0.1.
(b) The trial team decides to incorporate the information from the NOAH trial. They choose uniformly distributed priors for the event rates and are basing them on the 95%-confidence intervals from the trial, *i.e.*, $\pi_{T1}∼U_{\left[0.48,0.66\right]}$, $\pi_{C1}∼U_{\left[0.34,0.52\right]}$ and $\pi_{C2}∼U_{\left[0.5,0.7\right]}$. Since no data on HER2-negative patients treated with trastuzumab was available from the NOAH trial, they choose $\pi_{T2}∼U_{\left[0.5,0.7\right]}$. An optimal decision rule is then determined by incorporating the information obtained from the priors, which is $\left(c_{0}^{*},c_{1}^{*}\right)=\left(0.0822,0.0601\right)$.
(c) As in scenario (b), the trial team decides to incorporate the prior knowledge from the NOAH trial. However, they are more optimistic in regard of the treatment effect in the HER2-negative population and choose $\pi_{T2}∼U_{\left[0.5,0.8\right]}$. In this scenario, the optimal decision rule is $\left(c_{0}^{*},c_{1}^{*}\right)=\left(0.0915,0.0601\right)$.
(d) The trial team is unaware of the results of the NOAH trial and has no further information at hand. Hence, they decide to conduct the trial with an optimal decision threshold based on a non-informative uniform prior, *i.e.*, $\pi_{ij}∼U_{\left[0,1\right]}$, *i* = *T;C, j* = 1,2. The optimal decision rule is in this case $\left(c_{0}^{*},c_{1}^{*}\right)=\left(0.0807,0.1029\right)$.

We investigated the impact of the choice of the applied decision rule on the power of our design by a simulation study. Here, we chose *p_T_*_1_ = 0.6, *p_C_*_1_ = 0.45 and *p_C_*_2_ = 0.6. In order to investigate the sensitivity of the design with respect to parameter assumptions, we investigated two scenarios for the event rate of HER2-negative patients in the experimental group *p_T_*_2_, namely 0.65 and 0.7. In case *p_T_*_2_ = 0.65, the treatment effect in the total population would be ∆_0_ = 0.2·0.15 + 0.8·0.05 = 0.07 and hence below the relevance threshold *τ*_0_ making the selection of the total population unfavorable and thus less likely. For the scenario of a larger treatment benefit for HER2-negative patients, *i.e.*, *p_T_*_2_ = 0.7, ∆_0_ = 0.2·0.15+0.8·0.1 = 0.11 exceeds *τ*_0_ and thus a selection of the total population would be favorable here. For each scenario, 1,000,000 data sets were simulated (standard error for a rate of 0.5 equals 5·10^−4^). The results are displayed in Table 4.

First of all, we observe that decision rules (a) and (d) yielded the same results. This can be explained by the fact that, as in the simulation study in the previous section, the decision thresholds only slightly deviate from each other and this small difference did not have any influence on the interim decision. One can observe that decision rule (b) yields the largest overall power both in terms of rejecting the global null hypothesis *H*_0_ and rejecting either of the two local null hypothesis in both scenarios. In both scenarios, however, decision rule (c) achieves an only slightly worse performance as compared to decision rule (c). Both rules generally favor the selection of *G*_1_ at interim. This yields to a large advantage in power in scenario (A), where decision rule (c) performs slightly better than decision rule (b) when it comes to the detection of a treatment effect in *G*_1_. In scenario (A), the relatively high probability of 0.2361 for a futility stop may be a disadvantage of decision rules (a) and (d).

In scenario (B), decision rules (a) and (d) show the best performance when an effect in *G*_0_ is identified. However, these rules only slightly outperform decision rule (b). Here, decision rule (c) shows some minor disadvantage. since it yields a relatively high probability to select *G*_1_ only at interim, there is some lack in performance regarding the detection of the effect in *G*_0_. Again, decision rules (a) and (d) show the highest probability for a futility stop. However, the advantage is no longer that pronounced as in scenario (A).

Overall, we conclude that decision rule (b) is the one with the best overall performance in terms of power. Decision rule (c) comes close in case of scenario (A), but drops in performance in scenario (B). Decision rules (a) and (d) perform acceptably in scenario (B), but are outperformed by rules (b) and (c) in scenario (A).

## 7. Discussion

Adaptive enrichment designs, which include the option of selecting the most promising target population at interim, are a useful and powerful tool for the evaluation of targeted therapies. It can be assumed that alongside the rise of personalized medicine, adaptive enrichment designs will also experience an increasing importance in the near future. The decision which population to investigate during the second stage of such a design is a crucial one and, hence, it is important that the decision rule applied shows desirable properties in terms of successfully demonstrating a treatment effect.

In this article, we introduced an adaption of the design proposed by Jenkins *et al*. [7] in order to fit the setting of a binary outcome variable. For the situation of uncertainty about treatment effects, we derived and investigated optimal decision rules which take prior knowledge about event rates and trial characteristics, such as sample size and subgroup prevalence, into account. In our case, the derivation of these optimal decision rules was achieved by the use of standard computational software.

Within a simulation study and a clinical trial example, it was shown that the applied decision rule had substantial impact on the power of the trial. For the investigated parameter situations, optimal decision thresholds generally performed at least equally or better in terms of power as compared to a decision rule based on *ad hoc* assumptions.

In this article, we consider the specific setting of a single biomarker, one interim analysis and a binary outcome. In phase III trials in oncology, time-to-event variables are frequently used as primary endpoint. The presented optimal decision rules may then be applied if the interim decision is based on a short-term binary outcome, such as treatment response, and confirmatory analysis is performed for the time-to-event variable. In this case, even though the applied statistical tests are different, the decision framework and, accordingly, the applied decision rule, could adopt the approach developed in this work. Furthermore, the presented methodology can be easily transferred to a setting where the interim analysis occurs at an arbitrary time point. Consider the situation that not 4*n* but *tn* patients, with *t* > 2 being an arbitrary positive number, are enrolled during the trial with the interim analysis occurring after 2*n* patients. Then, solely the test statistics for the second stage have to be adapted and the weights for the combined test statistics have to be adjusted accordingly. The optimal decision thresholds for this situation would not change since they only depend on the data obtained from 2*n* patients during the first stage of the trial.

Throughout this article, we considered the situation of a bioassay which evaluates the biomarker with perfect sensitivity and specificity. However, in many cases this assumption may not hold true leading to patients being assigned to the wrong subgroup. It has been shown for a normally distributed outcome that the situation of imperfect classification leads to a potential downward bias in treatment effect estimates for the subgroup [14]. This has an immediate harmful impact on both the probability of correct interim decisions [14] and the power within an adaptive enrichment trial [15]. This worsening in performance in case of an imperfect bioassay can also be expected for the case of a binary outcome. Hence, it remains of paramount importance to carefully choose a bioassay with a sufficient accuracy when conducting an adaptive enrichment trial.

In summary, our investigations strongly highlight the importance to thoroughly evaluate the impact of the applied decision rule when planning a clinical trial with an adaptive enrichment procedure and yields to the recommendation to consider optimal decision thresholds as a possibility to increase the probability of successful trials and drug development programs.

## Figures and Tables

**Table 1:** Optimal decision thresholds $\left(c_{0}^{*},c_{1}^{*}\right)$ in case of a predictive prior, *i.e.*, $\pi_{T}{}_{1}\sim U_{0.3,0.6}$, $\pi_{T2},\pi_{C1},\pi_{C2}{∼}_{\mathrm{iid}}U_{\left[0.1,0.4\right]}$, in dependence of sample size *n* and subgroup prevalence π for relevance thresholds τ_0_ = 0*:*05, τ_1_ = 0.1.

*n*	π = 0.1	π = 0.2255	π = 0.5
20	(0.0908, −1.0000)	(0.0507, −0.4845)	(−0.0782, –0.1903)
40	(0.0688, −0.6247)	(0.0505, −0.1903)	(−0.0118, –0.0369)
60	(0.0623, −0.3885)	(0.0504, −0.0882)	(0.0098, 0.0138)
80	(0.0593, −0.2656)	(0.0503, −0.0369)	(0.0204, 0.0383)
100	(0.0576, −0.1903)	(0.0503, −0.0063)	(0.0266, 0.0525)
120	(0.0565, −0.1394)	(0.0502, 0.0138)	(0.0307, 0.0616)
140	(0.0557, −0.1029)	(0.0502, 0.0279)	(0.0336, 0.0679)
160	(0.0551, −0.0754)	(0.0502, 0.0383)	(0.0357, 0.0724)
180	(0.0547, −0.0540)	(0.0502, 0.0463)	(0.0374, 0.0758)
200	(0.0543, −0.0369)	(0.0501, 0.0525)	(0.0387, 0.0785)
300	(0.0531, 0.0138)	(0.0501, 0.0703)	(0.0426, 0.0861)
400	(0.0525, 0.0383)	(0.0501, 0.0785)	(0.0445, 0.0897)

**Table 2:** Optimal decision thresholds $\left(c_{0}^{*},c_{1}^{*}\right)$ in case of a predictive and prognostic prior, *i.e.*, $\pi_{T1}∼U_{\left[0.3,0.6\right]}$, $\pi_{C1}∼U_{\left[0.05,0.35\right]}$, $\pi_{T2}∼U_{\left[0.2,0.5\right]}$, $\pi_{C2}∼U_{\left[0.2,0.5\right]}$ in dependence of sample size *n* and subgroup prevalence π for relevance thresholds *τ*_0_ = 0.05, *τ*_1_ = 0.1.

*n*	π = 0.1	π = 0.2255	π = 0.5
20	(0.0915, −1.0000)	(0.0231, −0.8675)	(−0.1624, −0.3834)
40	(0.0690, −1.0000)	(0.0376, −0.3834)	(−0.0533, −0.1333)
60	(0.0622, −0.7077)	(0.0421, −0.2174)	(−0.0174, −0.0489)
80	(0.0591, −0.5060)	(0.0443, −0.1333)	(0.0003, −0.0071)
100	(0.0574, −0.3834)	(0.0455, −0.0826)	(0.0108, 0.0176)
120	(0.0562, −0.3008)	(0.0462, −0.0489)	(0.0176, 0.0337)
140	(0.0555, −0.2413)	(0.0468, −0.0250)	(0.0225, 0.0450)
160	(0.0549, −0.1964)	(0.0472, −0.0071)	(0.0261, 0.0533)
180	(0.0544, −0.1614)	(0.0475, 0.0067)	(0.0288, 0.0595)
200	(0.0541, −0.1333)	(0.0477, 0.0176)	(0.0310, 0.0645)
300	(0.0530, −0.0489)	(0.0484, 0.0494)	(0.0375, 0.0783)
400	(0.0524, −0.0071)	(0.0488, 0.0645)	(0.0407, 0.0846)

**Table 3:** Optimal decision thresholds $\left(c_{0}^{*},c_{1}^{*}\right)$ in case of a noninformative prior, *i.e.*, $\pi_{ij}{∼}_{\mathrm{iid}}U_{\left[0.1\right]}$, *i* = *T*, *C*, *j* = 0, 1, in dependence of sample size *n* and subgroup prevalence π for relevance thresholds *τ*_0_ = 0*:*05, *τ*_1_ = 0.1.

*n*	π = 0.1	π = 0.25	π = 0.5
20	(0.0572, 0.2066)	(0.0591, 0.1467)	(0.0610, 0.1239)
40	(0.0535, 0.1574)	(0.0546, 0.1239)	(0.0556, 0.1118)
60	(0.0523, 0.1393)	(0.0531, 0.1159)	(0.0538, 0.1078)
80	(0.0517, 0.1298)	(0.0523, 0.1118)	(0.0528, 0.1058)
100	(0.0514, 0.1239)	(0.0518, 0.1094)	(0.0523, 0.1046)
120	(0.0511, 0.1199)	(0.0515, 0.1078)	(0.0519, 0.1038)
140	(0.0510, 0.1171)	(0.0513, 0.1067)	(0.0516, 0.1033)
160	(0.0509, 0.1149)	(0.0511, 0.1058)	(0.0514, 0.1029)
180	(0.0508, 0.1132)	(0.0510, 0.1051)	(0.0513, 0.1025)
200	(0.0507, 0.1118)	(0.0509, 0.1046)	(0.0511, 0.1023)
300	(0.0505, 0.1078)	(0.0506, 0.1031)	(0.0508, 0.1015)
400	(0.0503, 0.1058)	(0.0505, 0.1023)	(0.0506, 0.1011)

**Table 4:** Probabilities to reject *H*_0_, $H_{0}^{\left(0\right)}$, $H_{0}^{\left(1\right)}$ and either $H_{0}^{\left(0\right)}$ $H_{0}^{\left(1\right)}$, or both, to select *G*_0_ and *G*_1_, and only *G*_0_ or *G*_1_, respectively, and to stop for futility at interim for treatment effect for the scenarios *p_T_*_2_ = 0.65 (A) and *p_T_*_2_ = 0.7 (B) and the proposed decision rules (a)–(d).

Scenario	Event	(a)	(b)	(c)	(d)
(A) *p_T_*_2_ = 0.65	reject *H*_0_	0.7564	**0.8901**	0.8882	0.7564
	reject $H_{0}^{\left(0\right)}$	**0.3615**	**0.3615**	0.2640	0.3615
	reject $H_{0}^{\left(1\right)}$	0.6874	0.8415	**0.8558**	0.6874
	reject either $H_{0}^{\left(0\right)}$, $H_{0}^{\left(1\right)}$, or both	0.7560	**0.8892**	0.8874	0.7560
	select *G*_0_ and *G*_1_	0.3226	**0.3587**	0.2610	0.3226
	select *G*_0_ only	0.0493	0.0132	0.0074	0.0493
	select *G*_1_ only	0.3919	0.5262	**0.6239**	0.3919
	stop for futility	**0.2361**	0.1018	0.1077	0.2361

(B) *p_T_*_2_ = 0.7	reject *H*_0_	0.8933	**0.9448**	0.9306	0.8933
	reject $H_{0}^{\left(0\right)}$	**0.8019**	0.8018	0.7107	0.8019
	reject $H_{0}^{\left(1\right)}$	0.6538	0.7738	**0.7900**	0.6538
	reject either $H_{0}^{\left(0\right)}$, $H_{0}^{\left(1\right)}$, or both	0.8932	**0.9445**	0.9301	0.8932
	select *G*_0_ and *G*_1_	0.6232	**0.7419**	0.6650	0.6232
	select *G*_0_ only	**0.1796**	0.0609	0.0462	0.1796
	select *G*_1_ only	0.0914	0.1431	**0.2200**	0.0914
	stop for futility	**0.1059**	0.0542	0.0688	0.1059

*n* = 400, *a* = 0.025; 1,000,000 simulation replications. The scenario(s) with the highest probability are marked in bold numbers.

## Supplementary Materials

Supplemenatry materials can be found at http://www.mdpi.com/1422-0067/16/05/10354/s1.

## Appendix

### A.1. Derivation of the Moments of the Effect Estimators

The rate difference estimators from the first stage of the trial are:

$${\hat{\Delta }}_{0}={\hat{p}}_{T0}^{I}-{\hat{p}}_{C0}^{I},\;{\hat{\Delta }}_{1}={\hat{p}}_{T1}^{I}-{\hat{p}}_{C1}^{I}$$

We can derive their expectations, variances and their covariance as follows:

$$\begin{array}{l} \;E\left[{\hat{\Delta }}_{0}\right]=\pi \left(p_{T1}-p_{C1}\right)+\left(1-\pi \right)\left(p_{T2}-p_{C2}\right),\;E\left[{\hat{\Delta }}_{1}\right]=p_{T1}-p_{C1},\\ \;\mathrm{Var}\left[{\hat{\Delta }}_{0}\right]=1/n^{2}\left(\mathrm{Var}\left[\sum_{i=1}^{\pi n}X_{i}^{T1}+\sum_{k=1}^{\left(1-\pi \right)n}X_{k}^{T2}\right]+\mathrm{Var}\left[\sum_{j=1}^{\pi n}X_{j}^{C1}+\sum_{l=1}^{(1-\pi )n}X_{l}^{C2}\right]\right)\\ \;=1/n^{2}\left(\sum_{i=1}^{\pi n}\mathrm{Var}\left[X_{i}^{T1}\right]+\sum_{k=1}^{\left(1-\pi \right)n}\mathrm{Var}\left[X_{k}^{T2}\right]+\sum_{j=1}^{\pi n}\mathrm{Var}\left[X_{j}^{C1}\right]+\sum_{l=1}^{(1-\pi )n}X_{l}^{C2}\right)\\ \;=1/n^{2}(\pi n(p_{T1}(1-p_{T1})+p_{C1}(1-p_{C1}))+(1-\pi )n(p_{T2}(1-p_{T2})+p_{C2}(1-p_{C2})))\\ \;=\frac{\pi (p_{T1}(1-p_{T1})+p_{C1}(1-p_{C1}))+(1-\pi )(p_{T2}(1-p_{T2})+p_{C2}(1-p_{C2}))}{n},\\ \;\mathrm{Var}\left[{\hat{\Delta }}_{1}\right]=\frac{p_{T1}\left(1-p_{T1}\right)+p_{C1}\left(1-p_{C1}\right)}{\pi n},\\ \mathrm{Cov}\left[{\hat{\Delta }}_{0},{\hat{\Delta }}_{1}\right]=\frac{1}{n}\cdot \frac{1}{\pi n}\left(\mathrm{Cov}\left[\sum_{i=1}^{\pi n}X_{i}^{T1}+\sum_{k=1}^{(1-\pi )n}X^{T2},\sum_{i=1}^{\pi n}X_{i}^{T1}\right]+\mathrm{Cov}\left[\sum_{j=1}^{\pi n}X_{j}^{C1}+\sum_{l=1}^{(1-\pi )l}X_{l}^{C2},\sum_{j=1}^{\pi n}X_{j}^{C1}\right]\right)\\ \;=\frac{1}{\pi n^{2}}\left(\sum_{i=1}^{\pi n}\mathrm{Cov}\left[X_{i}^{T1},X_{i}^{T1}\right]+\sum_{j=1}^{\pi n}\mathrm{Cov}\left[X_{j}^{C1},X_{j}^{C1}\right]\right)\\ \;=\frac{1}{\pi n^{2}}(\pi np_{T1}(1-p_{T1})+\pi np_{C1}(1-p_{C1}))\\ \;=\frac{p_{T1}\left(1-p_{T1}\right)+p_{C1}\left(1-p_{C1}\right)}{n} \end{array}$$

### A.2. Derivation of the Bayes Risk

The quadratic loss function for the considered scenario is:

$$\begin{array}{l} L:A\times {\left\{0,1\right\}}^{4}\to {\mathbb{R}}^{+},\\ L(a_{0},a_{1},\pi_{T1},\pi_{C1},\pi_{T2},\pi_{C2},)={\left[\pi (\pi_{T1}-\pi_{C1})+(1-\pi )(\pi_{T2}-\pi_{C2})-\tau_{0}\right]}^{2}\\ \;\cdot [1_{\left\{a_{0}=0,\pi (\pi_{T1}-\pi_{C1})+(1-\pi )(\pi_{T2}-\pi_{C2})\in (\tau_{0},\infty )\right\}}\left(a_{0},a_{1},\pi_{T1},\pi_{C1},\pi_{T2},\pi_{C2}\right)\\ \;+1_{\left\{a_{0}=1,\pi (\pi_{T1}-\pi_{C1})+(1-\pi )(\pi_{T2}-\pi_{C2})\in (-\infty ,\tau_{0}]\right\}}\left(a_{0},a_{1},\pi_{T1},\pi_{C1},\pi_{T2},\pi_{C2}\right)]\\ \;+{\left[\pi_{T1}-\pi_{C1}-\tau_{1}\right]}^{2}\\ \;\cdot [1_{\left\{a_{1}=0,\pi_{T1}-\pi_{C1}\in (\tau_{1},\infty )\right\}}\left(a_{0},a_{1},\pi_{T1},\pi_{C1},\pi_{T2},\pi_{C2}\right)\\ \;+1_{\left\{a_{1}=1,\pi_{T1}-\pi_{C1}\in (-\infty ,\tau_{1}]\right\}}\left(a_{0},a_{1},\pi_{T1},\pi_{C1},\pi_{T2},\pi_{C2}\right)], \end{array}$$

$\text{where}\;1_{\left\{a=i,x\in I\right\}}(a,x)=\{\begin{array}{cc} 1 & \mathrm{if}\;a=i,x\in I,\\ 0 & \text{else}, \end{array}\;a\in \{0,1\},I\subset \mathbb{R}$.

Since the binomial distribution can be assumed to be approximately normally distributed for rates not too close to zero or one and for sufficiently large sample size, it is valid to assume that $\left({\hat{\Delta }}_{0},{\hat{\Delta }}_{1}\right)$ is approximately bivariate normally distributed with:

$$\begin{array}{l} \left({\hat{\Delta }}_{0},{\hat{\Delta }}_{1}\right)|\pi_{T1},\pi_{C1},\pi_{T2},\pi_{C2}{∼}_{\text{approx}}N\left(\mu ,\sum \right),\\ \mu =\left(\mu_{0},\mu_{1},\right)=(\pi (\pi_{T1}-\pi_{C1})+(1-\pi )(\pi_{T2}-\pi_{C2}),\pi_{T1}-\pi_{C1}),\\ \sum =\left(\begin{array}{cc} \sum_{00} & \sum_{01}\\ \sum_{01} & \sum_{11} \end{array}\right)=\left(\begin{array}{cc} \frac{\pi (\pi_{T1}(1-\pi_{T1})+\pi_{C1}(1-\pi_{C1}))+(1-\pi )(\pi_{T2}(1-\pi_{T2})+\pi_{C2}(1-\pi_{C2})}{n} & \frac{\pi_{T1}(1-\pi_{T1})+\pi_{C1}(1-\pi_{C1})}{n}\\ \frac{\pi_{T1}(1-\pi_{T1})+\pi_{C1}(1-\pi_{C1})}{n} & \frac{\pi_{T1}(1-\pi_{T1})+\pi_{C1}(1-\pi_{C1})}{\pi n} \end{array}\right) \end{array}$$

In order to calculate the Bayes risk, we have to calculate the so-called likelihood which is the product of the densities of the priors and the conditional density of the effect estimators:

$$\begin{array}{l} p\left({\tilde{y}}_{0},{\tilde{y}}_{1},\pi_{T1},\pi_{C1},\pi_{T2},\pi_{C2}\right)=p\left({\tilde{y}}_{0},{\tilde{y}}_{1}|\pi_{T1},\pi_{C1},\pi_{T2},\pi_{C2}\right)\cdot p\left(\pi_{T1},\pi_{C1},\pi_{T2},\pi_{C2}\right)\\ \;\propto e^{-1/\left(2\left(1-\sum_{01}^{2}/\sum_{00}\sum {}_{11})\right)\right){\left[\left({\tilde{y}}_{0}-\mu_{0}\right)\right]}^{2}/\sum {}_{00}+{\left({\tilde{y}}_{1}-\mu_{1}\right)}^{2}/\sum {}_{11}-2\left({\tilde{y}}_{0}-\mu_{0}\right)\left({\tilde{y}}_{1}-\mu_{0}\right)\left({\tilde{y}}_{1}-\mu_{1}\right)\sum {}_{01}/\left(\sum {}_{00}\sum {}_{11}\right)}\\ \;\cdot \prod_{j=1,2}\prod_{i=T,C}1_{\pi_{ij}\in \left[a_{ij},b_{ij}\right]}\left(\pi_{ij}\right) \end{array}$$

For a given decision rule $d_{\left(c_{0},c_{1}\right)}$ we now obtain the Bayes risk:

$$\begin{array}{l} r\left(d_{c_{0},c_{1}}\right):=\int {}_{{\mathbb{R}}^{6}}L\left[d_{\left(c_{0},c_{1}\right)}\left({\tilde{y}}_{0},{\tilde{y}}_{1}\right),\pi_{T1},\pi_{C1},\pi_{T2},\pi_{C2}\right]\\ \;p\left({\tilde{y}}_{0},{\tilde{y}}_{1},\pi_{T1},\pi_{C1},\pi_{T2},\pi_{C2}\right)d{\tilde{y}}_{0}d{\tilde{y}}_{1}d\pi_{T1},d\pi_{C1},d\pi_{T2},d\pi_{C2}\\ \;\propto \int_{a_{C2}}^{b_{C2}}\int_{a_{T2}}^{b_{T2}}\int_{a_{C1}}^{b_{C1}}\int_{a_{T1}}^{b_{T1}}\int_{-\infty }^{\infty }\int_{-\infty }^{\infty }L\left[d_{\left(c_{0},c_{1}\right)}\left({\tilde{y}}_{0},{\tilde{y}}_{1}\right),\pi_{T1},\pi_{C1},\pi_{T2},\pi_{C2}\right]\\ \;\cdot p\left({\tilde{y}}_{0},{\tilde{y}}_{1}|\pi_{T1},\pi_{C1},\pi_{T2},\pi_{C2}\right)d{\tilde{y}}_{0}d{\tilde{y}}_{1}d\pi_{T1},d\pi_{C1},d\pi_{T2},d\pi_{C2} \end{array}$$

The above expression can be minimized by calculating the partial derivatives $d_{0}\left(c_{0}\right):=\partial r\left(d_{c_{0},c_{1}}\right)/\partial c_{0}$ and $d_{1}\left(c_{1}\right):=\partial r\left(d_{c_{0},c_{1}}\right)/\partial c_{1}$ and solving for the roots of the respective expressions, which are $c_{0}^{*}$ and $c_{1}^{*}$. In order to do so, we can split the above expression up into two summands *S*_0_ and *S*_1_ which only depend on the respective thresholds *c*_0_ and *c*_1_:

$$\begin{array}{l} S_{0}=\int_{a_{C2}}^{b_{C2}}\int_{a_{T2}}^{b_{T2}}\int_{a_{C1}}^{b_{C1}}\int_{a_{T1}}^{b_{T1}}[\int_{c_{0}}^{\infty }\int_{-\infty }^{\infty }{\left(\mu_{0}-\tau_{0}\right)}^{2}1_{\left\{\mu_{0}\leq \tau_{0}\right\}}(\mu_{0})\cdot p\left({\tilde{y}}_{0},{\tilde{y}}_{1}|\pi_{T1},\pi_{C1},\pi_{T2},\pi_{C2}\right)d{\tilde{y}}_{0}d{\tilde{y}}_{1}\\ \;+\int_{-\infty }^{c_{0}}\int_{-\infty }^{{}_{\infty }}{\left(\mu_{0}-\tau_{0}\right)}^{2}1_{\left\{\mu_{0}>\tau_{0}\right\}}(\mu_{0})\cdot p({\tilde{y}}_{0},{\tilde{y}}_{1}|\pi_{T1},\pi_{C1},\pi_{T2},\pi_{C2})d{\tilde{y}}_{0}d{\tilde{y}}_{1}]d\pi_{T1},d\pi_{C1},d\pi_{T2},d\pi_{C2},\\ S_{1}=\int_{a_{C2}}^{b_{C2}}\int_{a_{T2}}^{b_{T2}}\int_{a_{C1}}^{b_{C1}}\int_{a_{T1}}^{b_{T1}}[\int_{-\infty }^{\infty }\int_{c_{1}}^{\infty }{\left(\mu_{1}-\tau_{1}\right)}^{2}1_{\left\{\mu_{1}\leq \tau_{1}\right\}}(\mu_{1})\cdot p\left({\tilde{y}}_{0},{\tilde{y}}_{1}|\pi_{T1},\pi_{C1},\pi_{T2},\pi_{C2}\right)d{\tilde{y}}_{0}d{\tilde{y}}_{1}\\ \;+\int_{-\infty }^{\infty }\int_{-\infty }^{c_{1}}{\left(\mu_{1}-\tau_{1}\right)}^{2}1_{\left\{\mu_{1}>\tau_{1}\right\}}\left(\mu_{0}\right)\cdot p\left({\tilde{y}}_{0},{\tilde{y}}_{1}|\pi_{T1},\pi_{C1},\pi_{T2},\pi_{C2}\right)d{\tilde{y}}_{0}d{\tilde{y}}_{1}]d\pi_{T1},d\pi_{C1},d\pi_{T2},d\pi_{C2} \end{array}$$

$p\left({\tilde{y}}_{0},{\tilde{y}}_{1}|\pi_{T1},\pi_{C1},\pi_{T2},\pi_{C2}\right)$ is the density function of the bivariate normally distributed effect estimators with mean vector (*μ*_0_, *μ*_1_) and covariance matrix ∑. It is known that in order to obtain the marginal distribution of a multivariate normal distribution, one solely has to drop the irrelevant variables. Hence, we can simplify *S*_0_ and *S*_1_ to:

$$\begin{array}{l} S_{0}=\int_{a_{C2}}^{b_{C2}}{\int_{a_{T2}}^{b_{T2}}\int_{a_{C1}}^{b_{C1}}\int_{a_{T1}}^{b_{T1}}[\int_{c_{0}}^{\infty }{\left(\mu_{0}-\tau_{0}\right)}^{2}1_{\left\{\mu_{0}\leq \tau_{0}\right\}}(\mu_{0})e^{-{\left({\tilde{y}}_{0}-\mu_{0}\right)}^{2}/(2\sum_{00})}d\tilde{y}}_{0}\\ \;+\int_{-\infty }^{c_{0}}{\left(\mu_{0}-\tau_{0}\right)}^{2}1_{\left\{\mu_{0}>\tau_{0}\right\}}(\mu_{0})e^{-{\left({\tilde{y}}_{0}-\mu_{0}\right)}^{2}/(2\sum_{00})}d{\tilde{y}}_{0}]d\pi_{T1},d\pi_{C1},d\pi_{T2},d\pi_{C2},\\ S_{1}=\int_{a_{C2}}^{b_{C2}}\int_{a_{T2}}^{b_{T2}}\int_{a_{C1}}^{b_{C1}}\int_{a_{T1}}^{b_{T1}}[\int_{c_{1}}^{\infty }{\left(\mu_{1}-\tau_{1}\right)}^{2}1_{\left\{\mu_{1}\leq \tau_{1}\right\}}(\mu_{1})e^{-{\left({\tilde{y}}_{1}-\mu_{1}\right)}^{2}/(2\sum_{11})}d{\tilde{y}}_{1}\\ \;+\int_{-\infty }^{c_{1}}{\left(\mu_{1}-\tau_{1}\right)}^{2}1_{\left\{\mu_{1}>\tau_{1}\right\}}(\mu_{1})e^{-{\left({\tilde{y}}_{1}-\mu_{1}\right)}^{2}/(2\sum_{11})}d{\tilde{y}}_{1}]d\pi_{T1},d\pi_{C1},d\pi_{T2},d\pi_{C2} \end{array}$$

Now, since *S*_0_ does not depend on *c*_1_ and, analogously, *S*_1_ does not depend on *c*_0_, the partial derivatives *d*_0_ and *d*_1_ can be obtained in the following way using the Fundamental Theorem of Calculus:

$$\begin{array}{l} d_{0}(c_{0})=\frac{\partial S_{0}}{\partial c_{0}}=\int_{a_{C2}}^{b_{C2}}\int_{a_{T2}}^{b_{T2}}\int_{a_{C1}}^{b_{C1}}\int_{a_{T1}}^{b_{T1}}-{\left(\mu_{0}-\tau_{0}\right)}^{2}1_{\left\{\mu_{0}\leq \tau_{0}\right\}}(\mu_{0})e^{-{\left(c_{0}-\mu_{0}\right)}^{2}/(2\sum_{00})}\\ \;+{\left(\mu_{0}-\tau_{0}\right)}^{2}1_{\left\{\mu_{0}\leq \tau_{0}\right\}}(\mu_{0})e^{-{\left(c_{0}-\mu_{0}\right)}^{2}/(2\sum_{00})}d{\tilde{y}}_{0}d\pi_{T1}d\pi_{C1}d\pi_{T2}d\pi_{C2}\\ \;=\int_{a_{C2}}^{b_{C2}}\int_{a_{T2}}^{b_{T2}}\int_{a_{C1}}^{b_{C1}}\int_{a_{T1}}^{b_{T1}}\left({\left(\mu_{0}-\tau_{0}\right)}^{2}\cdot 1_{\left\{\mu_{0}\leq \tau_{0}\right\}}(\mu_{0}\right)e^{-{\left(c_{0}-\mu_{0}\right)}^{2}/(2\sum_{00})}d\pi_{T1}d\pi_{C1}d\pi_{T2}d\pi_{C2}\\ \;-\int_{a_{C2}}^{b_{C2}}\int_{a_{T2}}^{b_{T2}}\int_{a_{C1}}^{b_{C1}}\int_{a_{T1}}^{b_{T1}}\left({\left(\mu_{0}-\tau_{0}\right)}^{2}\cdot 1_{\left\{\mu_{0}\leq \tau_{0}\right\}}(\mu_{0}\right)e^{-{\left(c_{0}-\mu_{0}\right)}^{2}/(2\sum_{00})}d\pi_{T1}d\pi_{C1}d\pi_{T2}d\pi_{C2},\\ d_{1}(c_{1})=\frac{\partial S_{1}}{\partial c_{1}}=\int_{a_{C2}}^{b_{C2}}\int_{a_{T2}}^{b_{T2}}\int_{a_{C1}}^{b_{C1}}\int_{a_{T1}}^{b_{T1}}-{\left(\mu_{1}-\tau_{1}\right)}^{2}1_{\left\{\mu_{1}\leq \tau_{1}\right\}}(\mu_{1})e^{-{\left(c_{1}-\mu_{1}\right)}^{2}/(2\sum_{11})}\\ \;+{\left(\mu_{1}-\tau_{1}\right)}^{2}1_{\left\{\mu_{0}>\tau_{0}\right\}}(\mu_{1})e^{-{\left(c_{1}-\mu_{1}\right)}^{2}/(2\sum_{11})}d{\tilde{y}}_{0}d\pi_{T1}d\pi_{C1}d\pi_{T2}d\pi_{C2}\\ \;=\int_{a_{C2}}^{b_{C2}}\int_{a_{T2}}^{b_{T2}}\int_{a_{C1}}^{b_{C1}}\int_{a_{T1}}^{b_{T1}}\left({\left(\mu_{1}-\tau_{1}\right)}^{2}\cdot 1_{\left\{\mu_{1}>\tau_{1}\right\}}(\mu_{1}\right)e^{-{\left(c_{1}-\mu_{1}\right)}^{2}/(2\sum_{11})}d\pi_{T1}d\pi_{C1}d\pi_{T2}d\pi_{C2}\\ \;-\int_{a_{C2}}^{b_{C2}}\int_{a_{T2}}^{b_{T2}}\int_{a_{C1}}^{b_{C1}}\int_{a_{T1}}^{b_{T1}}\left({\left(\mu_{1}-\tau_{1}\right)}^{2}\cdot 1_{\left\{\mu_{1}\leq \tau_{1}\right\}}(\mu_{1}\right)e^{-{\left(c_{1}-\mu_{1}\right)}^{2}/(2\sum_{11})}d\pi_{T1}d\pi_{C1}d\pi_{T2}d\pi_{C2} \end{array}$$

The roots of *d*_0_ and *d*_1_ are the optimal decision thresholds $c_{0}^{*}$ and $c_{1}^{*}$, which can be determined by standard mathematical software such as, e.g., [20].
