# Supplementary material for: Optimal Decision Rules for Biomarker-Based Subgroup Selection for a Targeted Therapy in Oncology
Source: Int J Mol Sci. 2015 May 7;16(5):10354–75. doi: 10.3390/ijms160510354 (PMC4463650; doi:10.3390/ijms160510354)
Supplement: Supplementary file 1 [file ijms-16-10354-s001.zip › ijms-79388-supplementary final/Mathematica-Code_Optimal_rules.pdf]

```
(* Mathematica Code for determination of optimal decision thresholds
   [aC1,bC1]:        prior interval for event rates in G_1 in the control group

   [aC2,bC2]:        prior interval for event rates in G_2 in the control group

   [aT1,bT1]:        prior interval for event rates in G_1 in the treatment group

   [aT2,bT2]:        prior interval for event rates in G_2 in the treatment group

   n:                number of patients per group and stage

   prev:             prevalence of G_1

   tau0:             relevance threshold for G_0

   tau1:             relevance threshold for G_1

   c0:               decision threshold for G_0 to be optimized

   c1:               decision threshold for G_1 to be optimized

   g0[]:             derivative of the expected loss given G_0 is wrongly selected

   h0[]:             derivative of the expected loss given G_0 is wrongly not selected

   g1[]:             derivative of the expected loss given G_1 is wrongly selected

   h1[]:             derivative of the expected loss given G_1 is wrongly not selected

   f0[]:             derivative of the expected loss given a false decision concerning G_0
                     occurred

   f1[]:             derivative of the expected loss given a false decision concerning G_1
                     occurred

   b[]:              function that rounds the results on four digits and maps values smaller than
                           - 1 to -1 and values larger than 1 to 1, respectively

   opt0[]:           yields the root of f0, i.e. the optimal threshold c0*

   opt1[]:           yields the root of f1, i.e. the optimal threshold c1*

   opt[]:            yields [c0*,c1*]

Local adaptive method was applied for numerical integration with precision of 8 digits.

Secant method was applied for numerical root solving with precision of 3 digits.*)
```

```
g0[aC1_?NumericQ, bC1_?NumericQ, aC2_?NumericQ, bC2_?NumericQ,
  aT1_?NumericQ, bT1_?NumericQ, aT2_?NumericQ, bT2_?NumericQ,
  tau0_?NumericQ, n_?NumericQ, prev_?NumericQ, c0_?NumericQ] :=
 NIntegrate[-((prev * (pT1 - pC1) + (1 - prev) (pT2 - pC2) - tau0)) ^ (2) *
   Boole[((prev * (pT1 - pC1) + (1 - prev) (pT2 - pC2))) < tau0] * Exp[-(c0 -
         (prev * (pT1 - pC1) + (1 - prev) (pT2 - pC2)))^2 /
     (2 * ((prev (pT1 (1 - pT1) + pC1 (1 - pC1)) +
           (1 - prev) (pT2 (1 - pT2) + pC2 (1 - pC2))) / n))],
  {pT1, aT1, bT1}, {pC1, aC1, bC1}, {pT2, aT2, bT2}, {pC2, aC2, bC2},
  Method → "LocalAdaptive",
  PrecisionGoal → 8]

h0[aC1_?NumericQ, bC1_?NumericQ, aC2_?NumericQ, bC2_?NumericQ,
  aT1_?NumericQ, bT1_?NumericQ, aT2_?NumericQ, bT2_?NumericQ,
  tau0_?NumericQ, n_?NumericQ, prev_?NumericQ, c0_?NumericQ] :=
 NIntegrate[+((prev * (pT1 - pC1) + (1 - prev) (pT2 - pC2) - tau0)) ^ (2) *
   Boole[((prev * (pT1 - pC1) + (1 - prev) (pT2 - pC2))) > tau0] * Exp[-(c0 -
         (prev * (pT1 - pC1) + (1 - prev) (pT2 - pC2)))^2 /
     (2 * ((prev (pT1 (1 - pT1) + pC1 (1 - pC1)) +
           (1 - prev) (pT2 (1 - pT2) + pC2 (1 - pC2))) / n))],
  {pT1, aT1, bT1}, {pC1, aC1, bC1}, {pT2, aT2, bT2}, {pC2, aC2, bC2},
  Method → "LocalAdaptive",
  PrecisionGoal → 8]

g1[aC1_?NumericQ, bC1_?NumericQ, aT1_?NumericQ, bT1_?NumericQ,
  tau1_?NumericQ, n_?NumericQ, prev_?NumericQ, c1_?NumericQ] :=
 NIntegrate[-((pT1 - pC1) - tau1) ^ (2) * Boole[(pT1 - pC1) < tau1] * Exp[-(c1 -
         (pT1 - pC1))^2 / (2 * ((pT1 (1 - pT1) + pC1 (1 - pC1))) / (prev * n))],
  {pT1, aT1, bT1}, {pC1, aC1, bC1}, Method → "LocalAdaptive", PrecisionGoal → 8]

h1[aC1_?NumericQ, bC1_?NumericQ, aT1_?NumericQ, bT1_?NumericQ,
  tau1_?NumericQ, n_?NumericQ, prev_?NumericQ, c1_?NumericQ] :=
 NIntegrate[+((pT1 - pC1) - tau1) ^ (2) * Boole[(pT1 - pC1) > tau1] * Exp[-(c1 -
         (pT1 - pC1))^2 / (2 * ((pT1 (1 - pT1) + pC1 (1 - pC1))) / (prev * n))],
  {pT1, aT1, bT1}, {pC1, aC1, bC1}, Method → "LocalAdaptive", PrecisionGoal → 8]

f0[aC1_, bC1_, aC2_, bC2_, aT1_, bT1_, aT2_, bT2_, tau0_, n_, prev_, c0_] :=
 g0[aC1, bC1, aC2, bC2, aT1, bT1, aT2, bT2, tau0, n, prev, c0] +
  h0[aC1, bC1, aC2, bC2, aT1, bT1, aT2, bT2, tau0, n, prev, c0]

f1[aC1_, bC1_, aT1_, bT1_, tau1_, n_, prev_, c1_] :=
 g1[aC1, bC1, aT1, bT1, tau1, n, prev, c1] + h1[aC1, bC1, aT1, bT1, tau1, n, prev, c1]

b[x_] := Round[Sign[x] * Min[Abs[x], 1], 0.0001]

opt0[aC1_?NumericQ, bC1_?NumericQ, aC2_?NumericQ,
  bC2_?NumericQ, aT1_?NumericQ, bT1_?NumericQ, aT2_?NumericQ,
  bT2_?NumericQ, tau0_?NumericQ, n_?NumericQ, prev_?NumericQ] :=
 b[c0 /. FindRoot[f0[aC1, bC1, aC2, bC2, aT1, bT1, aT2, bT2, tau0, n, prev, c0],
    {c0, -1, 1, -2, 2}, PrecisionGoal → 3]]

opt1[aC1_?NumericQ, bC1_?NumericQ, aT1_?NumericQ,
  bT1_?NumericQ, tau1_?NumericQ, n_?NumericQ, prev_?NumericQ] :=
 b[c1 /. FindRoot[f1[aC1, bC1, aT1, bT1, tau1, n, prev, c1],
    {c1, -1, 1, -2, 2}, PrecisionGoal → 3]]
```

```
opt[pC1l_, pC1h_, pC2l_, pC2h_, pT1l_, pT1h_, pT2l_, pT2h_, tau0_, tau1_, n_,
  prev_] := {opt0[pC1l, pC1h, pC2l, pC2h, pT1l, pT1h, pT2l, pT2h, tau0, n, prev],
  opt1[pC1l, pC1h, pT1l, pT1h, tau1, n, prev]}

(* some example *)

opt[0.05, 0.35, 0.2, 0.5, 0.3, 0.6, 0.2, 0.5, 0.05, 0.1, 100, 0.25]

{0.0455, -0.0826}
```
